# Coeliac Disease: A Rare Cause of Myocardial Infarction With Non-Obstructive Coronary Arteries

**DOI:** 10.7759/cureus.38469

**Published:** 2023-05-03

**Authors:** Hasnaa Leghlimi, Hamza Chraibi, Nesma Bendagha, Aida Soufiani, Zoubida Tazi Mezalek

**Affiliations:** 1 Cardiovascular Surgery B Department, Ibn Sina Hospital, Mohammed V University, Rabat, MAR; 2 Cardiology A Department, Ibn Sina Hospital, Mohammed V University, Rabat, MAR; 3 Internal Medicine and Hematology Department, Ibn Sina Hospital, Mohammed V University, Rabat, MAR

**Keywords:** anticoagulation, thrombosis, cardiac magnetic resonance, myocardial infarction, coeliac disease

## Abstract

Myocardial infarction with non-obstructive coronary arteries (MINOCA) poses a diagnostic dilemma. Identifying the underlying etiology is essential to ensuring appropriate management. Cardiac magnetic resonance (CMR) is a valuable tool that can aid clinicians for that purpose. Coeliac disease (CD) is characterized by hypercoagulability and a thrombotic state and represents an exceptional cause of MINOCA. We report the case of a 28-year-old woman who presented with chest pain. The diagnosis of non-ST-elevation MI was obtained based on ECG abnormalities and elevated troponin levels. Coronary angiography was normal. CMR showed late gadolinium enhancement in the lateral left ventricular wall, confirming the diagnosis of MINOCA. A duodenal biopsy allowed the diagnosis of CD. Anticoagulation and a gluten-free diet proved beneficial, with a good outcome after a five-year follow-up. This case highlights the essential role of CMR in MINOCA investigations and the importance of thorough etiological assessment in young patients with no cardiovascular risk factors.

## Introduction

Myocardial infarction with non-obstructive coronary arteries (MINOCA) is a challenging entity whose prevalence recently rose due to the systematic use of angiography following acute coronary syndrome [1]. Recent improvements in intracoronary imaging, such as optical coherence tomography and intravascular ultrasound, allowed for a better understanding of this pathology [2]. Cardiac magnetic resonance imaging (CMR) is a valuable, non-invasive diagnostic tool that plays a pivotal role in the diagnostic workup of MINOCA [3].

In this paper, we report the case of a 28-year-old woman who presented with non-ST-elevation acute myocardial infarction (MI), in whom CMR proved essential in the diagnosis of MINOCA and led us to the correct etiology through specific investigations.

## Case presentation

**Figure 1:**
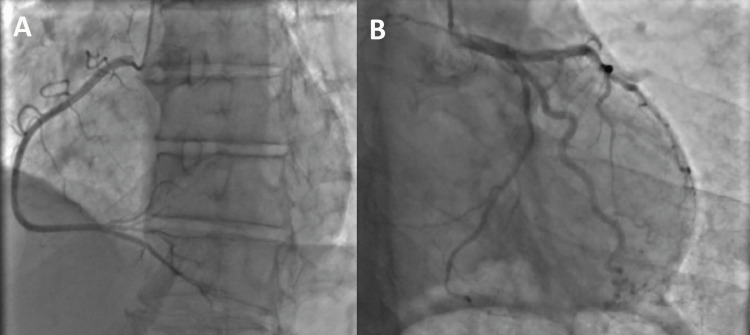
A coronary angiogram of our patient shows normal coronary arteries. A: left anterior oblique view of the right coronary artery and its branches; B: right anterior oblique view of the left coronary artery and its branches

A 28-year-old woman with a history of multiple spontaneous abortions and chronic abdominal discomfort, and no cardiovascular risk factors presented three days after an episode of moderate-intensity chest pain and dizziness. She had no history of drug abuse, such as cocaine and amphetamines. She was admitted to the cardiology department. Initial physical examination was normal, but the patient was underweight with a body mass index of 18 kg/m². An electrocardiogram at admission found ST depression in inferior leads, slight ST elevation in lateral leads, and Q waves in V4-V5-V6 leads. Standard lab tests were normal, but there was moderate troponin elevation, confirming the diagnosis of non-ST-elevation MI. A transthoracic echocardiogram showed mild global hypokinesia with regional strain anomalies in the lateral wall. The ejection fraction was calculated at 54%. Coronary angiography was normal, confirming the diagnosis of MINOCA (Figure 1).

**Figure 2:**
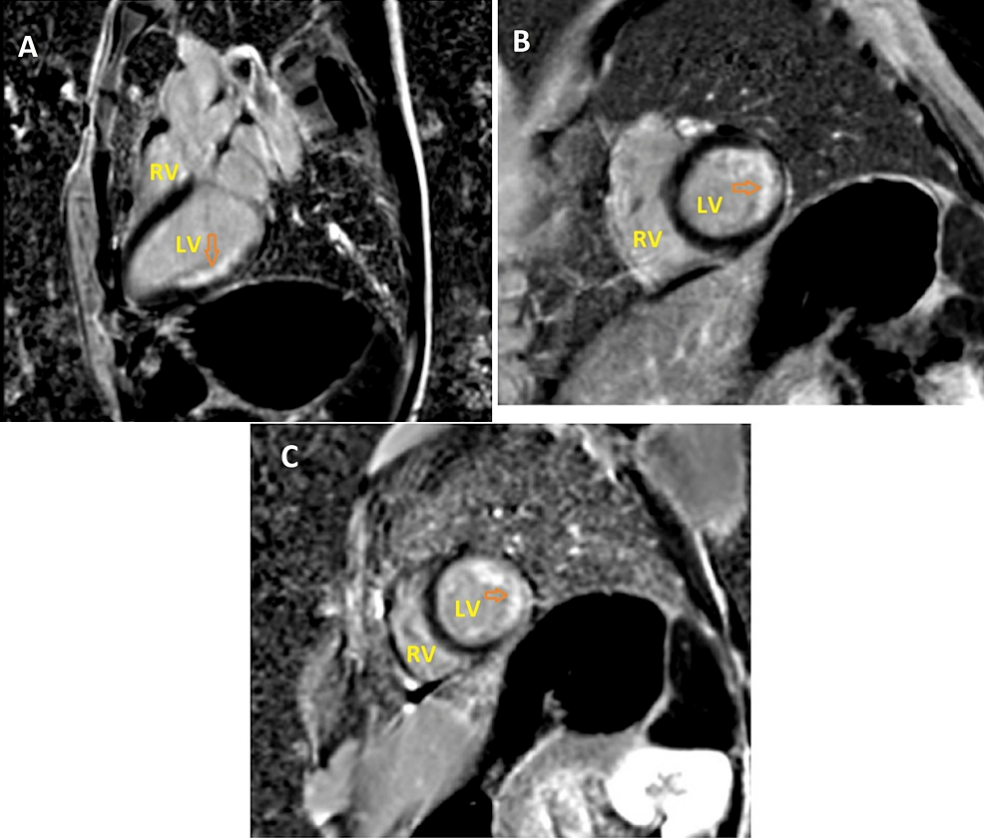
CMR of our patient, late gadolinium enhancement sequence, long axis (A), median short axis (B), and apical short axis (C) views showing near transmural LGE at the medial and apical segments of the left ventricle’s inferolateral wall (orange arrows). LV: left ventricle; RV: right ventricle.

CMR showed a lateral infarction with a near-transmural late gadolinium enhancement distribution (Figure 2). A malabsorption syndrome was suspected because of the low body weight. Blood tests revealed a protein C and protein S deficiency. Tissue transglutaminase antibodies were positive, and a duodenal biopsy confirmed the diagnosis of coeliac disease (CD). Lifetime anticoagulation (oral acenocoumarol with INR control) and a gluten-free diet were started. After a five-year follow-up, the patient is well with no symptomatic recurrence, but she refused control imaging examinations.

## Discussion

MINOCA cases account for 5% to 15% of acute MI [4]. According to the 2020 European Society of Cardiology Guidelines for the Management of Non-ST-Elevation MI, the diagnosis of MINOCA is made in patients with acute MI fulfilling the following criteria [3]: 1) Acute MI (according to the Fourth Universal Definition of Myocardial Infarction); 2) Non-obstructive coronary arteries on angiography (no stenosis ≥ 50%); 3) No specific alternate diagnosis for the clinical presentation.

This definition excludes myocarditis and Takotsubo syndrome from the final diagnosis of MINOCA. Both the ESC and the American Heart Association have recognized the central role of CMR as the gold standard exam in the positive and differential diagnosis of patients with MINOCA. In particular, the 2020 ESC guidelines recommend CMR in all MINOCA cases with no obvious underlying cause (Class IB). CMR provides diagnosis through tissue characterization by showing a subendocardial ischemic late gadolinium enhancement (LGE) pattern in the LGE sequences that can be optimized using novel LGE 3D sequences [5]. CMR also eliminates differential diagnoses, notably myocarditis, Takotsubo syndrome, and other cardiomyopathies. After reviewing angiography findings, specific ischaemic diagnoses should be investigated, such as coronary artery spasm (15.5%), thrombophilia (14%) with coronary emboli, thrombi, or microvascular disease. For this purpose, intracoronary imaging and functional testing are essential [6]. Even after all these investigations, the etiology remains unknown in 8%-25% of patients. 

The underlying etiology of each patient must be precisely determined, as many causes benefit from specific treatments. In situ thrombus formation, then, lysis may be a potential mechanism, resulting in a normal coronary angiography. According to a recent review, about 14% of patients with MINOCA may have an abnormality detected on thrombophilia screening [7].

Our patient had thrombophilia, which is evident from her history of repeated spontaneous abortions, and we were able to relate her MINOCA to CD because of her low weight and chronic abdominal discomfort.

Thromboembolic events in CD have previously been reported. Multiple constituents may explain this thrombophilic tendency; we can cite vitamin B12 or folate deficiency, hyperhomocysteinemia, enzyme mutations, and protein C and S deficiency as a consequence of vitamin K deficiency. The immunologic theory has been at the forefront of many papers lately. Greater exposure to autoantigens, such as phospholipids or epitopes, results in the production of autoantibodies, which play a major role in CD-related thromboembolic events and represent promising targets for future preventive therapy [8].

The CD should be suspected in cases of unexplained thrombotic manifestations (similar to our patient), even if digestive signs are absent [9,10]. CD may therefore be included as a risk factor in cardiovascular disease risk prediction models. The QRISK3 algorithm already includes autoimmune conditions such as systemic lupus erythematosus [11].

In the 2020 ESC guidelines, patients with an initial diagnosis of MINOCA should be treated and followed up according to the guidelines of the specific etiology [2]. Patients with MINOCA seem to have a better prognosis than patients with obstructive coronary stenosis, but large cohort clinical trials are needed for further exploration of these specificities [12].

For CD, risk factors for thrombosis must be investigated, corrected, or even prescribed as thromboembolic prophylaxis [12]. Early diagnosis and treatment with a gluten-free diet are essential to reducing mortality and comorbidities. In our patient’s case, anticoagulation with oral acenocoumarol and gluten-free products proved beneficial with a good long-term outcome.

## Conclusions

CMR plays a major role in the diagnosis of MINOCA, as it can provide a precise diagnosis of a broad range of heart diseases and eliminate differential diagnoses.

Treatment of MINOCA is largely cause-oriented, so the etiologic investigation is of paramount importance. This report illustrates the benefit of advanced investigations to diagnose specific etiologies of MINOCA, especially thromboembolic causes such as CD.
